# Repetitive Transcranial Magnetic Stimulation of Dorsolateral Prefrontal Cortex Affects Performance of the Wisconsin Card Sorting Task during Provision of Feedback

**DOI:** 10.1155/2008/143238

**Published:** 2008-02-27

**Authors:** Ji Hyun Ko, Oury Monchi, Alain Ptito, Michael Petrides, Antonio P. Strafella

**Affiliations:** ^1^Montreal Neurological Institute, McGill University, Montréal, PQ, Canada H3A 2B4; ^2^PET Imaging Centre, Centre for Addiction and Mental Health, University of Toronto, Toronto, ON, Canada M5T 1R8; ^3^Functional Neuroimaging Unit, Geriatric’s Institute, University of Montréal, Montréal, PQ, Canada H3W 1W5; ^4^Toronto Western Research Institute and Hospital, University of Toronto, Toronto, ON M5T 2S8, Canada

## Abstract

Early functional neuroimaging studies of tasks evaluating executive processes, such as the Wisconsin card sorting task (WCST), only assessed trials in blocks that may contain a large amount of different cognitive processes. More recently, we showed using event-related fMRI that the dorsolateral prefrontal cortex (DL-PFC) significantly increased activity during feedback but not matching periods of the WCST, consistent with its proposed role in the monitoring of information in working memory. Repetitive transcranial magnetic stimulation (rTMS) is a method that allows to disrupt processing within a given cortical region and to affect task performance for which this region is significantly solicited. Here we applied rTMS to test the hypothesis that the DL-PFC stimulation influences monitoring of working memory without interfering with other executive functions. We applied rTMS to the right DL-PFC and the vertex (control site) in different time points of the WCST. When rTMS was applied to the DL-PFC specifically during the period when subjects were receiving feedback regarding their previous response, WCST performance deteriorated, while rTMS did not affect performance during matching either when maintaining set or during set-shifting. This selective impairment of the DL-PFC is consistent with its proposed role in monitoring of events in working memory.

## 1. INTRODUCTION

There is considerable evidence that damage to the prefrontal cortex impairs performance on cognitive set-shifting tasks [1–3]. In one such task, the Wisconsin card sorting task (WCST), the subject has to match, over successive trials, a test card to one of four reference cards based on a matching rule that the subject acquires on the basis of feedback provided after each matching response. Patients with prefrontal lesions are often impaired in shifting the principle of matching when the feedback provided indicates that a cognitive shift in mental set is required. Functional neuroimaging studies support these observations [4–6]. In a recent study, conducted with functional magnetic resonance imaging (fMRI), we demonstrated differential activation of different parts of the prefrontal cortex during the performance of the WCST. In particular, we were able to show that the dorsolateral prefrontal cortex (DL-PFC) was engaged when feedback was provided [4]. This selective engagement of the mid-DL-PFC during the provision of feedback after each matching response by the subject is consistent with the proposed role of this part of the prefrontal cortex in the monitoring of events in working memory [7–9]. Neuroimaging studies, however, suffer from the limitation that they provide neuronal correlates of cognitive performance and cannot determine a causal relation between observed brain activity and cognitive performance [10, 11]. Thus the specific functional relevance of the DL-PFC in monitoring the feedback provided during the performance of set-shifting tasks remains to be established.

Here we have used repetitive transcranial magnetic stimulation (rTMS) to examine this issue. The application of rTMS to an area of cortex that, at a particular point in time, is actively involved in the processing of task-relevant information should cause performance to decline [12–14]. In other words, rTMS acts as a “virtual lesion” producing a temporary interruption of processing [15]. In the present study, we tested the hypothesis that rTMS of the human DL-PFC influences monitoring of the information held in the working memory without interfering with other executive functions. To test this specific hypothesis, we used a computerized version of the WCST [4] in which different stages of task performance can be isolated. We applied rTMS to the right DL-PFC and over a control site (the vertex) in three different ways: at the beginning of the feedback period, at the beginning of the matching response period, and independently of task timing. Our previous functional neuroimaging study had indicated the involvement of the DL-PFC during the provision of feedback, but not during the matching response. To further strengthen our findings, we also added a control task (Figure 3(b)) that only required matching to a twin card.

## 2. MATERIALS AND METHODS

Ten healthy subjects (19–33 years) participated in the study after having given written informed consent. All subjects were right-handed according to the Edinburgh handedness inventory [16], they had no previous personal or family history of neurological or psychiatric disorders and were not taking any medication at the time of experiments. The experiments were approved by the Research Ethics Committee of the Montreal Neurological Institute and Hospital. Figure 2 displays an overview and timing of the experimental setup.

### 2.1. Cognitive task

Subjects were trained for 30 minutes on the WCST before the rTMS sessions. Prior to the training sessions, the subjects were instructed to perform as well as they could. During the WCST, four reference cards and one matching card were presented on a computer screen (Figure 3(a)). On each trial, the subjects had to match a test card to one of the four reference cards according to one of three rules: shape, number, or color. The currently appropriate rule for classification is found by trial and error based on the 3-second positive or negative feedback that is provided immediately after each matching decision. The rule for classification changed randomly after the subject answers correctly on six consecutive trials. In the control task, the matching card was identical to one of the reference cards so that the subject simply selected the identical card and did not have to find an appropriate rule for classification as in the WCST (Figure 3(b)). Subjects performed the card-sorting tasks in six different rTMS sessions (2 × 3 design). Five-minute breaks were given in between sessions. Each session lasted six minutes.

### 2.2. Frameless stereotaxy system

**Figure 1:**
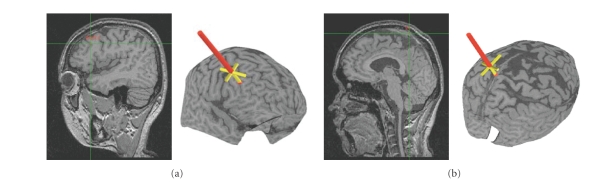
TMS coil was located over (a) the right DL-PFC (*X* = 45, *Y* = 33, *Z* = 25) or (b) the vertex (control) (*X* = 0, *Y* = −35, *Z* = 80). The positioning of the TMS coil over these locations, marked on the native MRI, was performed with the aid of a frameless stereotaxic system.

In order to target the DL-PFC and vertex (control site) in all our subjects (Figure 1), we used a procedure that takes advantage of the standardized stereotaxic space of Talairach and Tournoux [17] and frameless stereotaxy [18, 19]. A high-resolution MRI of the subjects' brain was acquired and transformed into standardized stereotaxic space using the algorithm of Collins et al. [20]. The coordinates selected for the right DL-PFC (*X* = 45, *Y* = 33, *Z* = 25) were based on a previous functional activation study that yielded increased activity during the feedback period [4]. Of note, in this study, we stimulated the DL-PFC located in the right hemisphere because this side appeared to be more consistently and robustly activated [4]. The control stimulation site (i.e., vertex region, *X* = 0, *Y* = −35, *Z * = 80) was also chosen based on its lack of activation during performance of the WCST in these previous studies.

The Talairach coordinates were converted into each subject's native MRI space using the reverse native-to-talairach transformation [18]. The positioning of the TMS coil over these locations, marked on the native MRI (Figure 1), was performed with the aid of a frameless stereotaxic system (Rogue Research, Montreal, Canada).

### 2.3. TMS protocol

Repetitive TMS was carried out with the Magstim high-speed magnetic stimulator (Magstim, UK) using a figure-eight coil. The coil was held in a fixed position over the stimulation sites by a mechanical arm. It was positioned so that magnetically induced current under the coil flowed in a posterior-anterior direction. Stimulus intensities, expressed as a percentage of the maximum stimulator output, were set at 110% of the resting motor threshold (RMT). RMT was defined as the lowest stimulus intensity able to elicit, in the contralateral first dorsal interosseous (FDI) muscle, 5 motor evoked potentials (MEPs) of at least 50 uV amplitude in a series of 10 stimuli delivered over the right primary motor cortex at intervals longer than 5 seconds. MEPs were recorded from the FDI muscle with Ag∖Cl surface electrodes fixed on the skin with a belly-tendon montage. The EMG signal was filtered (10 Hz–1 kHz bandpass), digitized at 2 kHz, and displayed on a computer screen [19].

**Figure 2:**
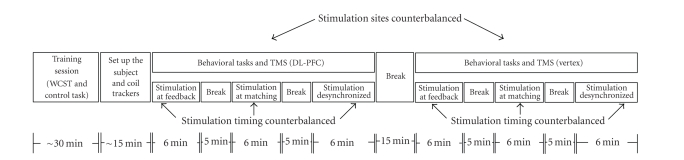
Timeline of the experimental setup. All subjects were trained for approximately 30 minutes at the beginning of the experiment. After registering the subjects' anatomical land marks to their structural MRIs, the subjects performed 6 minutes of the behavioral tasks while rTMS was administered at DL-PFC or vertex (control) in three different timing conditions. The orders of stimulation sites and timings were counterbalanced. The behavioral tasks consisted of WCST and control task.

**Figure 3:**
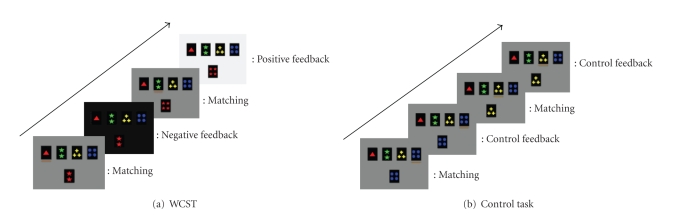
Behavioral tasks. (a) WCST: the four cards shown on the top in the computer screen are reference cards, and the card on the bottom is the test card. The subjects could move a yellow bar which was displayed under the reference cards by pressing the left button of a mouse with their index finger. Pressing the right button with the middle finger confirmed the selection of the card followed by negative or positive feedback. The subjects had to find out the rule of classification (color, shape, and number) by trial and error. (b) Control task: the test card was identical to one of the reference cards. The rest was the same as WCST.

**Figure 4:**
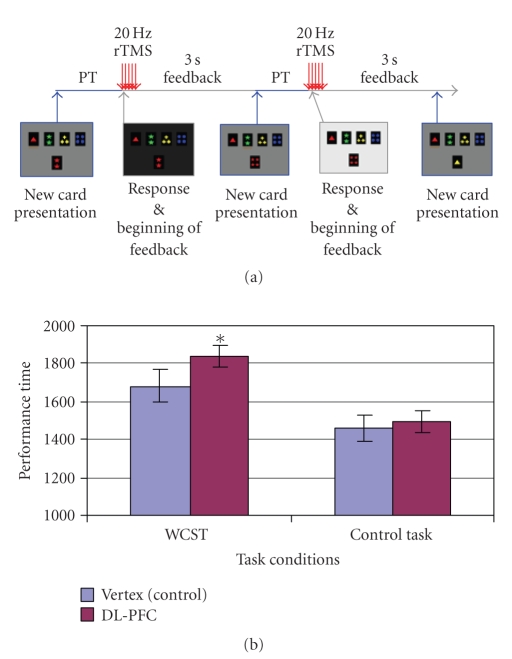
(a) rTMS at the beginning of feedback: while the subject performed the WCST or control task, rTMS was applied over the right DL-PFC or vertex at the beginning of receiving feedback. (b) DL-PFC stimulation during the feedback phase of the WCST increased performance time (PT) compared to the vertex stimulation (^*^ ** *P* = .023; two-tailed). No stimulation effect was observed in the control task.

**Figure 5:**
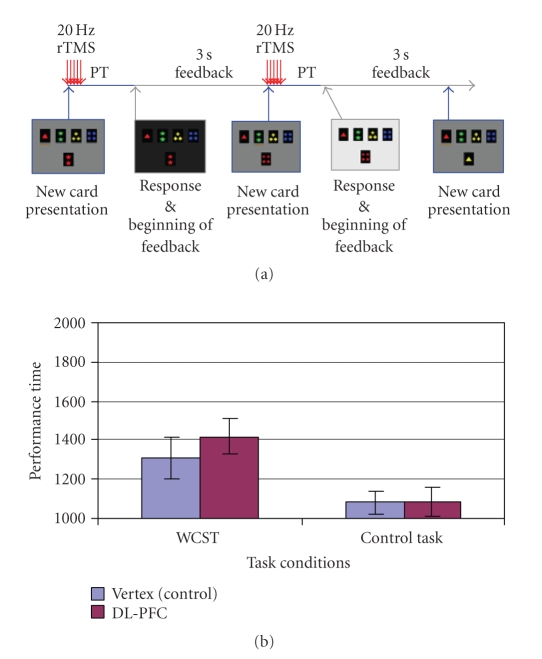
(a) rTMS at the beginning of matching: while the subject performed the WCST or control task, rTMS was applied over the right DL-PFC or vertex at the beginning of matching. (b) DL-PFC stimulation during the matching phase of WCST or control task had no effect on PT compared to the vertex stimulation.

**Figure 6:**
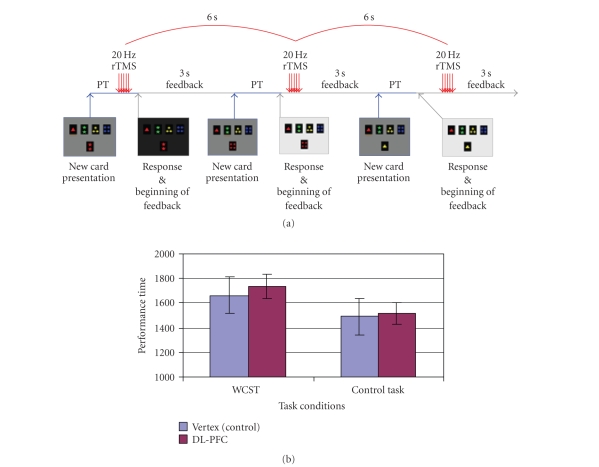
(a) Desynchronized rTMS: while the subject performed the WCST or control task, rTMS was applied over the right DL-PFC or vertex at every 6 seconds which was desynchronized with the tasks. (b) Desynchronized DL-PFC stimulation had no effect on PT compared to the vertex stimulation.

Three rTMS blocks (6 minutes each) were applied to the right DL-PFC and the vertex during the WCST and control task (Figure 2). Each block was separated by a 5-minute interval. In each block, 5 pulse trains of 250-millisecond duration were delivered at a stimulation frequency of 20 Hz with between-train interval dependent on the subject's performance time (PT) (i.e., 4 to 6 second). For each block, rTMS was delivered either *(block-1)* at the beginning of each feedback period (number of trials: 72.05 ± 0.75) (Figure 4), *(block-2)* at the beginning of each matching period (number of trials: 74.15 ± 1.19) (Figure 5), or *(block-3)* every 6 second regardless of the moment in the task (i.e., desynchronized condition) (number trials: 75.53 ± 2.14) (Figure 6). This last paradigm was applied in order to investigate whether the rTMS effect was timing dependent (i.e., block-1 and -2) or not (block-3). Block order was counterbalanced across subjects and performed on the same day (Figure 2). The stimulation parameters followed safety guidelines for rTMS [21].

### 2.4. Data analysis

PT and error rate were calculated. Each subject's PT and error rate were averaged within each condition (stimulation site, timing, and task). PT was measured from the presentation of the test card to the subject's response, that is, the selection of a reference card (Figures 4, 5 and 6).

Repeated-measures ANOVA was used to compare the effect of the two different stimulation sites, the three timings of stimulation, and the two different tasks on PT.

The paired samples *t*-test (two-tailed) was used to compare the mean PT and error rate in the WCST between the DL-PFC and vertex stimulations during the three different rTMS timing conditions (rTMS during feedback, during matching, and desynchronized). The mean PT for the control task was also compared in the same manner. Data are presented as mean ± SE. All statistical analysis was performed using SPSS 13.0 for Windows (SPSS Inc., USA).

## 3. RESULTS

TMS intensity was 58.4 ± 2.8%. There was no significant difference between numbers of trials among different blocks. Repeated-measures ANOVA on PT revealed a significant main effect of different tasks (WCST versus control; *F*(1,9) = 71.3; *P* < .001) confirming that the WCST was more demanding than the control task. There was also a significant main effect of stimulation timing on PT (beginning of feedback versus beginning of matching versus desynchronized; *F*(2,18) = 23.845; *P* < .001) indicating that the timing of stimulation, overall, was an important factor influencing task performance more than stimulation site (DL-PFC versus vertex; *F*(1,9) = 2.516; *P* = .147). A significant interaction effect was observed between tasks and stimulation site (*F*(1,9) = 7.642; *P* = .022) indicating that stimulation site affected PT differently depending on which task was used.

To test the effect of different stimulation sites within each task and stimulation timing condition, a paired *t*-test (two-tailed) was performed. When comparing DL-PFC versus vertex during the WCST, PT increased significantly when rTMS was delivered at the beginning of the feedback period (DL-PFC = 1840.04 ± 87.18 ms, Vertex = 1682.46 ± 61.23 ms; *t*(9) = 2.727; *P* = .023) (Figure 4). Further analysis revealed that the magnitude of impairment did not correlate with intensity of TMS (*r* = −0.063; *P* = .863). No changes in PT were observed when rTMS was given at the beginning of the matching period (DL-PFC = 1419.19 ± 107.48 ms, Vertex = 1309.87 ± 88.07 ms; *t*(9) = 1.382; *P* = .200) (Figure 5) nor when it was desynchronized with task performance (DL-PFC = 1739.13 ± 148.26 ms, Vertex = 1659.70 ± 98.24 ms; *t*(9) = 0.944; *P* = .370) (Figure 6). When comparing DL-PFC versus vertex during the control task, rTMS did not induce significant changes in PT either during the feedback (DL-PFC = 1491.66 ± 65.47 ms, Vertex = 1459.48 ± 59.60 ms; *t*(9) = 0.669; *P* = .521) (Figure 4), matching (DL-PFC = 1084.92 ± 62.15 ms, Vertex = 1080.26 ± 77.11 ms; *t*(9) = 0.074; *P* = .943) (Figure 5), or desynchronized (DL-PFC = 1517.38 ± 147.72 ms, Vertex = 1490.91 ± 90.36 ms; *t*(9) = 0.314; *P* = .760) conditions (Figure 6).

The repeated-measures ANOVA on error rate did not show any significant main effect of task conditions, stimulation timing, or the sites of stimulation, nor significant interaction effects except when comparing DL-PFC and vertex at the beginning of feedback which came close to significance. More specifically, the results obtained when performing a paired *t*-test on the error rates between DL-PFC and vertex stimulation during the WCST wereat the beginning of the feedback (DL-PFC = 6.10 ± 1.71, Vertex = 3.28 ± 1.16; *t*(9) = 2.120; *P* = .063); at the beginning of matching (DL-PFC = = 4.79 ± 1.04, Vertex = 4.86 ± 1.27; *t*(9) = −0.057; *P* = .956); during the desynchronized condition (DL-PFC = 5.21±0.83, Vertex = 3.56 ± 0.51; *t*(9) = 1.941; *P* = .084).

## 4. DISCUSSION

The present study demonstrated that when rTMS was applied to the DL-PFC specifically during the period when the subject was receiving feedback regarding his/her matching response, performance of the WCST deteriorated. It appeared that the effect of rTMS was significantly timing dependent. In fact, rTMS-induced interference of DL-PFC affected performance specifically during the receiving of feedback (Figure 4), but not during the matching response (Figure 5) nor when the interference was desynchronized with specific stages of the WCST (Figure 6).

This observation of a selective rTMS-induced impairment in task performance during specific timing of a task has already been reported in the literature in relation to several of the tasks and cortical areas stimulated. For instance, rTMS of the medial frontal cortex affected task switching and at the time of response set switching when delivered before or at time of response selection [10, 22]. Similarly, rTMS affected DL-PFC depending on whether this area, at a particular point in time, is actively involved in processing task relevant information [11, 23].

The selective rTMS-induced impairment in WCST performance of DL-PFC during the receiving of feedback is in accordance with imaging, lesion, and neurophysiological investigations. In a previous fMRI study, Monchi et al. [4] have shown that DL-PFC is engaged when the subject is receiving feedback during the WCST. That is, the period when monitoring of information held in working memory, as demonstrated by lesion studies in monkeys, is critical [8, 24]. This specific involvement has also been confirmed with neuronal recordings from DL-PFC in monkeys during a WCST analog which have shown the activation of DL-PFC cells during monitoring and use of feedback information. A large population of DL-PFC cells were strongly engaged in assessing behavioral outcome/feedback [25].

Interestingly, while rTMS induced selective impairment in WCST performance, it did not affect error rate very significantly. This observation is consistent with previous work by Wagner et al. [26] who, stimulating the DL-PFC, observed no significant effect on error making during the WCST. There are two potential alternatives that could explain these findings.

The first explanation is that error making may be influenced by a different prefrontal area. In fact, lesions of DL-PFC in monkeys have shown impairment in monitoring of information but did not compromise maintenance of information and set shifting per se [8, 9, 24], which presumably may influence errors during set-shifting tasks. Set shifting from a previously relevant to a new response mode engages a more ventral area of the PFC (i.e., ventrolateral PFC) [4] and is impaired by lesioning of this area [24, 27]. Another cortical area that may also have a relevant role is the medial PFC which can influence error trials during performance-monitoring processes [25].

A second explanation, considering the fact that rTMS-induced error trials have been reported less frequently in relation to different tasks and cortical area stimulated [10, 22, 23, 28], it may also be that rTMS parameters (e.g., intensity, frequency, and unilateral stimulation) used so far in different studies have not been strong enough to induce a complete “virtual lesion.” Against the latter hypothesis, however, stands the fact that the magnitude of selective impairment in WCST performance observed in this study did not correlate with intensity of TMS which at least excludes a possible relationship between intensity and effect on performance.

While our study provides some insights over the debate regarding the role of DL-PFC during set-shifting tasks, overall it emphasizes the importance of rTMS in delineating the functional relevance of neuronal correlates of performance observed during neuroimaging studies [10, 11]. In other words, our results suggest that just because a cortical area (i.e., DL-PFC) is functionally activated during the course of an executive task [4], it may not necessarily play the same critical and essential role during the whole task, and that rTMS may be a useful tool to complement fMRI in order to infer functionality of a cortical region of the human brain.

To date, the neural mechanisms underlying executive processes are still poorly understood, even less are the mechanisms by which rTMS interferes with cortical information processing and induces such a “temporary lesion.” It is believed that the rTMS-induced “noise” into neural processes may, perhaps, be the consequence of a stimulation-induced synchronization of neuronal firing disrupting active processing in the underlying cortex [15, 29]. A valid alternative, however, may also be represented by a suppression in cortical excitability (lasting up to 1 second) observed following short trains of rTMS at 20 Hz [30] or induced abnormality in the release of prefronto-striatal dopamine [19].

The latter is suggested by the contribution of the striatum and role played by dopamine during the performance of tasks requiring executive processes. Indeed, studies of dopamine depletion in nonhuman primates suggest a possible involvement of striatal dopamine in set-shifting tasks [31, 32] while other neuroimaging studies have proposed that changes in striatal dopamine levels can modulate certain set-shifting processes [33] and that level of cognitive impairment may be dependent on the level of dopamine depletion [34].

Whatever the rTMS mechanisms may be, the ultimate outcome appears to be a transient interruption of the specific normal cortical processing (i.e., provision of feedback) in a restricted area of the prefrontal cortex (i.e., DL-PFC).
